# Genome analysis of *Ranavirus frog virus 3* isolated from American Bullfrog (*Lithobates catesbeianus*) in South America

**DOI:** 10.1038/s41598-019-53626-z

**Published:** 2019-11-20

**Authors:** Marcelo Candido, Loiane Sampaio Tavares, Anna Luiza Farias Alencar, Cláudia Maris Ferreira, Sabrina Ribeiro de Almeida Queiroz, Andrezza Maria Fernandes, Ricardo Luiz Moro de Sousa

**Affiliations:** 1 Universidade de São Paulo (USP), Department of Veterinary Medicine, Pirassununga, 13635-900 Brazil; 2 Technical University of Denmark, The National Veterinary Institute, Bygning 204, Lyngby, Denmark; 3 Agência Paulista de Tecnologia dos Agronegócios (APTA), Fisheries Institute, Sao Paulo, 05001-900 Brazil

**Keywords:** Molecular evolution, Phylogenetics, Taxonomy, Viral epidemiology, Viral evolution

## Abstract

Ranaviruses (family *Iridoviridae*) cause important diseases in cold-blooded vertebrates. In addition, some occurrences indicate that, in this genus, the same virus can infect animals from different taxonomic groups. A strain isolated from a *Ranavirus* outbreak (2012) in the state of Sao Paulo, Brazil, had its genome sequenced and presented 99.26% and 36.85% identity with samples of *Frog virus 3* (FV3) and *Singapore grouper iridovirus* (SGIV) ranaviruses, respectively. Eight potential recombination events among the analyzed sample and reference FV3 samples were identified, including a recombination with *Bohle iridovirus* (BIV) sample from Oceania. The analyzed sample presented several rearrangements compared to FV3 reference samples from North America and European continent. We report for the first time the complete genome of *Ranavirus* FV3 isolated from South America, these results contribute to a greater knowledge related to evolutionary events of potentially lethal infectious agent for cold-blooded animals.

## Introduction

Among the major viral pathogens, worldwide distributed and recent history, *Ranavirus* (Rv) is highlighted, on which, studies in South America remain limited. Rv are part of the family *Iridoviridae* that is divided into five genera, of which three are considered more relevant by infectious severity in aquatic and semi-aquatic animals: *Lymphocystivirus*, *Megalocytivirus* and Rv. They are enveloped and unenveloped viruses, showing double-stranded DNA whose genome ranges from 103 to 220 kbp. Their viral particles have a diameter of approximately 150–200 nm and can be found in both fresh and salt water^1,2^.

Rv are considered emerging pathogens due to the recent increase in the incidence of infections, their expanding geographic range, and an increase in the range of susceptible hosts^3^. They are able to infect ectothermic vertebrates from three different taxonomic groups: amphibians, fishes and reptiles^4^. In addition, some occurrences indicate that, in this genus, the same virus can infect animals from different taxonomic groups^5^.

Rv infection is recognized as one of the main pathologies of economic and ecological importance in cold-blooded vertebrates^1,4^ and furthermore these viruses are suspected to be partially responsible for the global declines in amphibian populations^4^.

In the host, Rv cause systemic disease involving multiple internal organs, mainly hematopoietic tissues, kidneys, liver, spleen and gastrointestinal tract, causing focal hemorrhages and generalized necrosis^1,6^. Rv have also been shown to induce characteristic clinical signs of apoptosis in host cells, such as chromatin condensation and DNA fragmentation^4^.

The present work aimed to characterize the Rv genome from South America, evaluating the phylogeny, identity, recombination and rearrangement characteristics, using sequences of different Rv species as reference.

## Methods

This research project was approved by the ethics committee on the use of vertebrate animals of the Faculty of Animal Science and Food Engineering (Universidade de São Paulo), case number 1629310316. All experiments were performed in accordance with relevant guidelines and regulations.

In the present study, eight samples of liver, spleen and kidney collected from American Bullfrogs (*Lithobates catesbeianus*) in an Rv outbreak that occurred in 2012 in a frog farm located in the state of Sao Paulo, Brazil, confirmed by PCR and nucleotide sequencing using primers M151, M152, M153 and M154, were pooled and grinded to viral isolation in cell culture (BF-2 cells - *bluegill fry* ATCC^®^ CCL-91^TM^) following the protocol recommended by the World Organisation For Animal Health (OIE)^7^.

Extraction of viral DNA from cell culture was performed using the QIAamp DNA Mini Kit (Qiagen, Germany), following the manufacturer’s instructions. The total DNA underwent quality analysis through 2% agarose gel electrophoresis and quantification by fluorescence in a Qubit^®^ 2.0 Fluorometer (ThermoFisher Scientific, USA). The DNA was subjected to purification steps using Agencourt AMPure XP magnetic bead (Beckman Coulter, USA) and was quantified again by fluorescence using the Qubit^®^ 2.0 Fluorometer (Thermo Fisher Scientific, USA).

DNA fragment libraries were prepared with 50 ng of purified DNA using a Nextera DNA sample preparation kit and sequenced using an Illumina^®^ MiSeq System (Illumina, USA). After quality control of the sequence reads using Trim Galore (https://github.com/FelixKrueger/TrimGalore)^8^ de novo assembly using SPAdes produced contigs of 105 kilobase pairs with an G + C content of 54.98%. All annotations were performed manually with the aid of the software ORF finder (https://www.ncbi.nlm.nih.gov/orffinder/) to predict the location of coding open reading frames, using the genome of Rv *Frog virus 3* (FV3) isolate wt-FV3 (Genbank accession No. AY548484), and 94 potentials Open Reading Frames (ORFs) were noted. The virus genome sequence in this study was submitted to GenBank under accession number MH351268.

For phylogenetic characterization, identity analysis, recombination detection and rearrangement analyzes of the genome under analysis, complete genome sequences of 19 members from *Iridoviridae* family representing all species of Rv that have the genome available were retrieved from GenBank (Table 1).

**Table 1:** *Ranavirus* genomic sequences used for phylogenetic reconstruction, identity analysis, recombination detection and rearrangement analyzes with indication of the Rv species, host, country and respective access codes in GenBank.

Ranaviruses species	Host	Country	GenBank access code
EHNV-like	*Perca fluviatilis*	Australia	FJ433873
EHNV-like	*Perca fluviatilis*	Australia	NC_028461
ECV-like	*Ameiurus nebulosus*	Hungary	KT989885
ECV-like	*Ameiurus nebulosus*	Hungary	KT989884
ATV-like	*Ambystoma tigrinum*	United States	KR075874
ATV-like	*Ambystoma tigrinum*	United States	KR075886
ATV-like	*Ambystoma mexicanum*	United States	KR075872
CMTV-like	*Testudo kleinmanni*	Germany	KP266743
CMTV-like	*Sander lucioperca*	Finland	KX574341
CMTV-like	*Pelophylax klepton esculentus*	Denmark	MF538627
CMTV-like	*Pelophylax ridibundus*	Netherlands	MF004271
BIV-like	*Limnodynastes ornatos*	Australia	KX185156
FV3-like	*Hoplobatrachus tigerinus*	China	AF389451
FV3-like	*Rana grylio*	China	JQ654586
FV3-like	*Oophaga pumilio*	Netherlands	MF360246
FV3-like	Frog (host not informed)	United States	KJ175144
FV3-like	*Lithobates pipiens*	United States	AY548484
SGIV-like	Host not informed	Uninformed origin	AY521625
SGIV-like	Host not informed	Uninformed origin	NC_006549

EHNV- Epizootic haematopoietic necrosis virus; ECV - European catfish virus; ATV - Ambystoma tigrinum virus; CMTV - Common midwife toad virus; BIV - Bohle iridovirus; FV3 - Frog virus 3; SGIV - Singapore grouper iridovirus.

The DNA sequences of Rv were aligned using the software MAFFT version 7 with default settings (https://mafft.cbrc.jp/alignment/server/)^9^. Phylogenetic reconstruction was generated by the Neighbor-Joining (NJ) method, Kimura 2-parameter model, with bootstrap nodal support for 1000 pseudoreplicates in MEGA software, version 6.0^10^.

Pairwise identity scores using the complete genome and in parallel the major capsid protein (MCP) of Rv were calculated using Sequence Demarcation Tool (SDT)^11^ with default settings. The MCP gene is highly conserved among the different species of Rv, which makes it suitable for the presumptive diagnosis in infected animals through conventional PCR reaction^12^.

The recombination analysis among complete genomes of Rv FV3 was carried using RDP4 program version 4.95 in default settings, with *p*-value of <0.05. Genome rearrangement was assessed using Mauve 2.4.0^13^, with match seed weight of 15, gap open score of −400 and gap extend score of −30.

## Results

### Phylogenetic analysis

**Figure 1:**
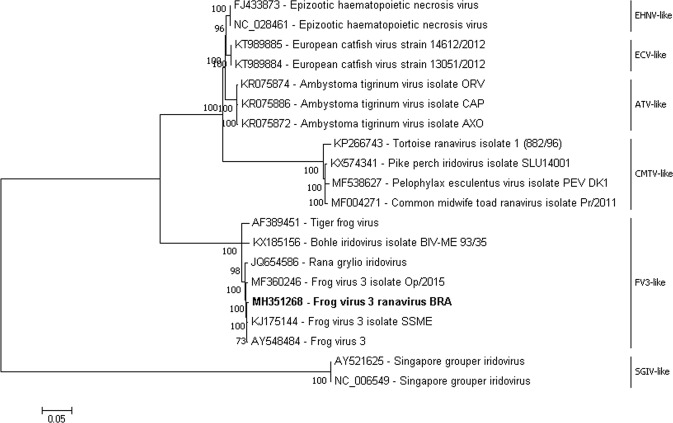
Phylogram representing phylogenetic reconstruction using the genomes of the different species of *Ranavirus* available in GenBank (ATV-like *Ambystoma tigrinum virus*, CMTV-like *Common midwife toad virus*, EHNV-like *Epizootic haematopoietic necrosis virus*, ECV-like *European catfish virus*, FV3-like *Frog virus 3*, SGIV-like *Singapore grouper iridovirus*). Bootstrap values higher than 70% for 1000 pseudoreplicates are showed at the nodes. The sequence obtained and analyzed in the present study is highlighted. GenBank accession numbers are shown on the tree. The scale bar represents the phylogenetic distance among sequences.

The sample sequenced in this study (MH351268) was grouped in the Rv FV3-like clade, presenting a *bootstrap* value of 100% with sequences of the same viral species (Fig. 1).

### Analysis of MCP gene identity among different species of *Ranavirus*

**Figure 2:**
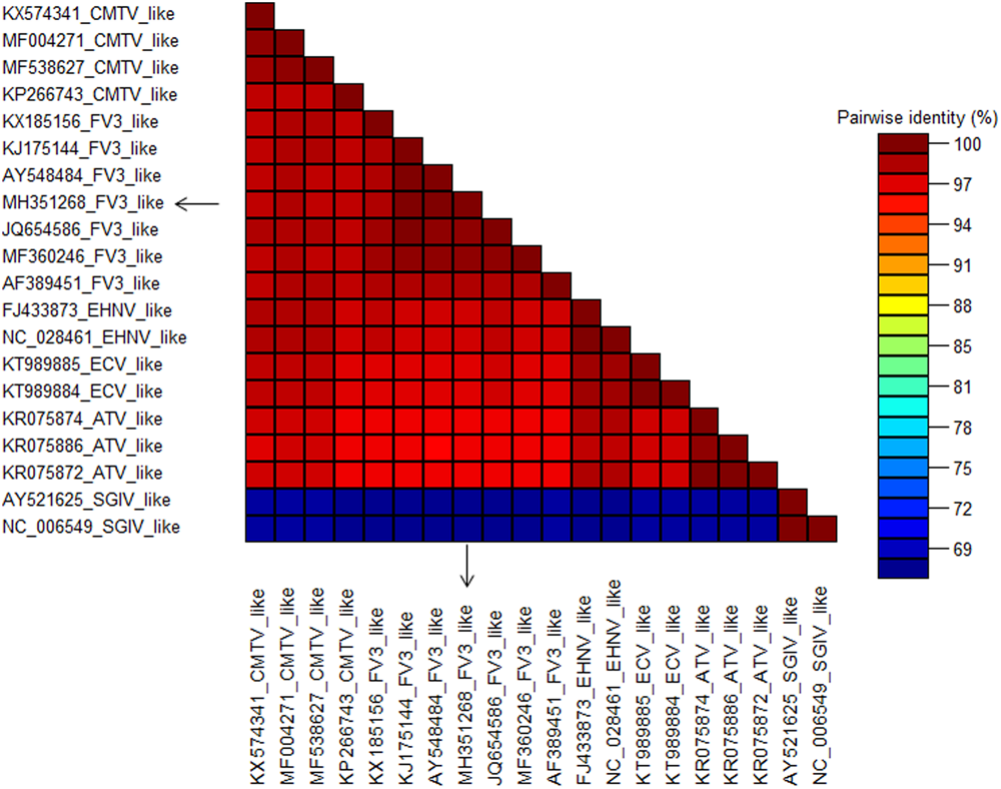
Pairwise nucleotide sequence identities of the MCP gene among different species of *Ranavirus*. The arrow in black indicates the sample obtained and analyzed in this study.

Analyzing the MCP gene, the nucleotide identity among the samples under analysis was greater than 94% for all Rv species, except for *Singapore grouper iridovirus* (SGIV) isolates that showed identity below 69% (Fig. 2).

### Identity analysis among ranaviruses genomes

The sequenced Rv showed the highest nucleotide identity of the genome (99.26%) with North American FV3 isolates (AY548484 and KJ175144) and only 36.85% with SGIV isolates (AY521625 and NC_006549). The overall mean of identity among the genomes was 62.01% (Supplementary Fig. S1).

### Recombination analysis among *Ranavirus frog virus 3* genomes

Eight potential recombination events were identified among the sample analyzed in this study (MH351268) and reference samples of FV3 (Supplementary Fig. S2, Table 2).

**Table 2:** Recombinant regions among the South American genome of FV3 and reference samples.

Sample	Recombinant regions	Number of ORFs in recombination	Main products encoded within these recombinant regions
JQ654586 (major)	1398-1730	1	Myristylated membrane protein (ORF 2).
MF360246 (major)	15817-18629	4	AAA-ATPase (ORF 17).
AY548484 (major)	21970-29766	5	Putative D5 family NTPase/ATPase (ORF 24).
MF360246 (major)	55030-89859	33	DNA polymerase (ORF 60); putative interleukin-1 beta convertase precursor (ORF 64); possible membrane associated motif in LPS-induced tumor necrosis factor alpha factor (ORF 73); putative ATPase-dependet protease (ORF77); cytosine DNA methyltransferase (ORF 81).
KJ175144 (major) and JQ654586 (minor)	62856-66431	4	Putative phosphotransferase (ORF 56).
AY548484 (major) and AF389451 (minor)	76073-78001	2	Ribonucleoside diphosphate reductase beta subunit (ORF 65).
AY548484 (major)	81146-82918	4	Possible membrane associated motif in LPS-induced tumor necrosis factor alpha factor (ORF 73).
KX185156 (minor)	96170-96375	1	TPR domain protein (ORF 86).

Several hypothetical proteins are within the recombinant regions among the analyzed samples.

### Rearrangement analysis among *Ranavirus frog virus 3* genomes

Comparing the South American sample (MH351268) with North American samples AY548484 and KJ175144 (99.26% identity), the former presented the insertion of a hypothetical protein in ORFs 3, 8, 15, 54, 58, 62, 66 and 92.

The ORFs 31, 44, 47 and 70 showed inversion of reading in South American sample “right” and in North American samples “left”. Analyzing the ORF 18, the South American sample showed “left” reading and the North American samples “right” reading.

The ORFs 33 “left” and 34 “right” (neurofilament triplet h1-like protein) presented truncation, differentiating into two different ORFs, while North American samples do not have this characteristic. The ORFs 22 and 77 showed shortening compared to reference samples. On the other hand, the ORFs 37, 38 and 41 presented inversion of reading plus shortening.

Several deletions of ORFs were identified in the sample under analysis compared to reference samples: deletion of an ORF between ORFs 31 and 32, two OFRs between ORFs 43 and 45, an ORF between ORFs 45 and 46, an ORF between ORFs 60 and 61, two ORFs between ORFs 64 and 65, one ORF between ORFs 83 and 84, one ORF between ORFs 88 and 89, and one ORF after ORF 94. As in the KJ175144 sample, the sample under analysis showed a combination of two ORFs in one (ORF 48) when compared to sample AY548484.

**Figure 3:**
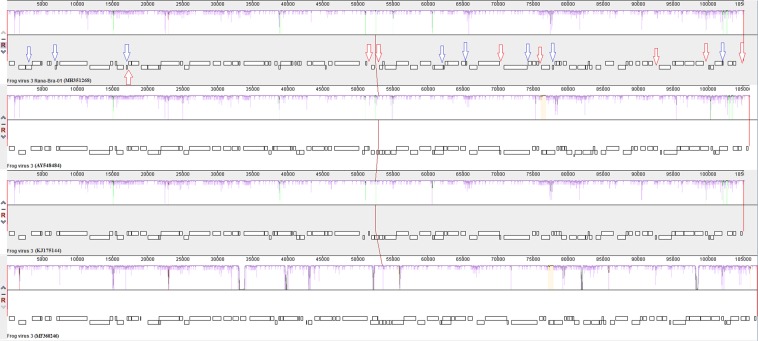
Analysis of rearrangement among samples of Rv FV3 from South America, North America and Europe. Arrows in blue indicate insertion of ORFs and arrows in red indicate deletion of ORFs in the South American FV3 sample, compared to reference samples.

The genome of the FV3 sample from the Netherlands (KJ175144) was also compared to the South American sample, due to the large recombinant region. This sample also showed several deletions and insertions within the genome compared to the sample under analysis. A high degree of rearrangement between Rv was evidenced (Fig. 3).

### Analysis of rearrangement among different species of *Ranavirus*

**Figure 4:**
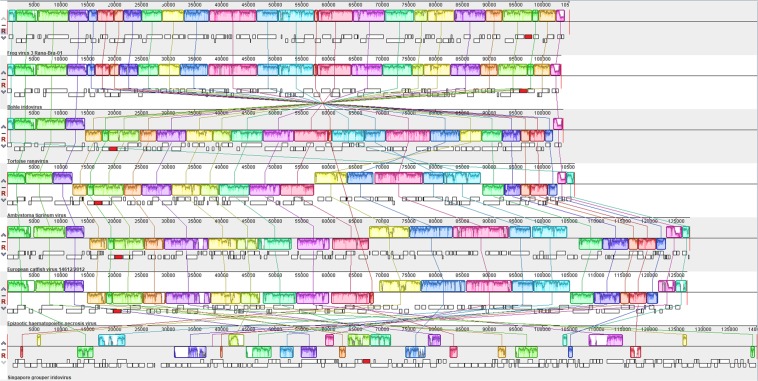
Analysis of rearrangement among different species of *Ranavirus*. The MCP gene, conserved among the different RV species, is highlighted in red. *Frog virus 3* Rana-Bra-01 (MH351268); Bohle iridovirus (KX185156); Tortoise ranavirus (KP266743); *Ambystoma tigrinum virus* (KR075872); European catfish virus (KT989885); *Epizootic haematopoietic necrosis virus* (FJ433873); *Singapore grouper iridovirus* (AY521625).

When comparing the genomes of different species of Rv, the only species that shows complete collinearity with the FV3 genome analyzed in this study was the genome of *Bohle Iridovirus* (BIV), all other species showed genomic inversions in some region of the genome compared to the analyzed FV3 sample (Fig. 4).

## Discussion

The number of nucleotides, percentage of G + C and number of ORFs of the genome analyzed are in accordance with those described in the literature for the genotyped virus^14,15^.

The nucleotide phylogenetic reconstruction grouped the South American Rv genome in the FV3-like clade, presenting a 100% bootstrap value with genomic samples of the same viral species. In an intriguing way, KX185156 sample, derived from Rv of the BIV species, entered the clade of FV3-like presenting ≥98% of bootstrap value. BIV was the virus-causing epizootic outbreak with 100% mortality of captive tilapias of the specie *Oreochromis mossambicus*, within a period of 60 days in Australia^16^.

Similar to this work, other recent studies have also demonstrated the insertion of BIV-like into the clade of FV3-like^17^. The identity between the genome under analysis and the KX185156 (BIV) sample was 94.85%, a relatively high percentage because it is a different species within the same viral genus.

When the complete nucleotide sequences of the MCP gene (1392 bp) of different Rv species were analyzed, identities were greater than 94.00%, except for SGIV isolates, which showed identity below 69% compared to other species within the same genus. Similar results were described by Waltzek *et al*.^18^, comparing nucleotide sequences of the MCP gene between the SGIV and FV3 species isolated from sturgeon (*Scaphirhynchus albus*); the percentage of identity between samples was 69.4% and among FV3 and other species of Rv (FV3, BIV, CMTV, EHNV, ECV and ATV), the identity for the gene was >94.1%.

Through the acquisition of genes via recombination, members of viral families that present dsDNA adapt to their respective hosts^19^. Recombinant events are recognized as driving forces in the generation of pathogenic viruses and as a form of viral adaptation to new hosts^20–23^. All samples used for the recombination analysis (JQ654586, KX185156, AF389451, MF360246, KJ175144 and AY548484) showed recombination events (major parent or minor parent *p* < 0.05) with the South American sample of Rv FV3 in some region of the genome.

We also documented recombination (minor parent) between Rv FV3 and BIV. Events of recombination among Rv of different species were also reported by Claytor *et al*.^24^, who detected recombination between FV3 and CMTV viruses, generating a pathogenic chimeric Rv denominated RCVZ2 that infect amphibians. The authors isolated and analyzed strains from a commercial facility in different years (1998 and 2006), suggesting that the virus evolution occurred in the locality and that one or more CMTV virus genes have been rearranged through processes of recombination.

Some studies have identified naturally occurring recombination events among Rv isolates^25,26^. It is suggested that the inherent frequency of high recombination of Rv leads to a sharp rearrangement of the organization of the viral genome, presenting a diversified genomic organization as a reflex^27^. Rv that show greater sequence divergence can also show lower sequence collinearity over time derived from increased recombination of the viral genome^28^. International trade of live animals such as the American bullfrog (*Lithobates catesbeianus*) and cultured fish has been associated with the emergence of these viruses^29–31^.

Rearrangement and recombination were identified between FV3 samples collected in North America (AY548484 and KJ175144), Europa (MF360246) and South America (MH3512168), highlighting the role that the international trade in farmed animals may have played in the global dissemination of highly pathogenic Rv. Recombination in double-stranded DNA viruses (dsDNA) has been reported by some researchers, such as herpesvirus, poxvirus and bacteriophages^32–34^.

High rates of recombination of Rv *in vitro* have been suggested, however the mechanism and significance of these genomic changes remain unknown^27,28,35^. Thus, more investigations are needed to elucidate the mechanisms and meanings of Rv evolution, associating any relation with its ecology, host interaction and pathogenesis^28^.

Infectious diseases in insects, mammals, marsupials, amphibians, reptiles and fish are not yet fully understood. Globally, combating infectious diseases is a major goal of public and veterinary health efforts. Since Rv infects a wide variety of ecologically and economically important hosts, understanding the evolution of these viruses, including the importance of the genome rearrangements found among isolates in relation to host specificity and viral evolution will help predict and eventually prevent epizootics^36^.

This study adds and reinforces the potential recombination among Rv. Although this study provides information on recombination and rearrangements of Rv, future works should focus on understanding this genomic variability among these viruses and its relationship to viral ecology, host range, and pathogenesis.

In conclusion, we report here for the first time the genome of Rv FV3 isolated from South America. Our results contribute to a better understanding of the characteristics of viral agents potentially associated with severe outbreaks in cold-blooded vertebrates.

## Supplementary information

Supplementary Figures

## Data Availability

The *Ranavirus Frog Virus 3*-like DNA sequence analysed in the present investigation was deposited in the GenBank database under the accession code MH351268.
