# Supplementary material for: Genome analysis of Ranavirus frog virus 3 isolated from American Bullfrog (Lithobates catesbeianus) in South America
Source: Sci Rep. 2019 Nov 20;9:17135. doi: 10.1038/s41598-019-53626-z (PMC6868289; doi:10.1038/s41598-019-53626-z)

Genome analysis of *Ranavirus frog virus 3* isolated from American Bullfrog (*Lithobates catesbeianus*) in South America

Authors

Marcelo Candido[1,*], Loiane Sampaio Tavares[1], Anna Luiza Farias Alencar[2], Cláudia Maris Ferreira[3], Sabrina Ribeiro de Almeida Queiroz[1], Andrezza Maria Fernandes[1], and Ricardo Luiz Moro de Sousa[1]

Institutions

[1]Universidade de São Paulo (USP), Department of Veterinary Medicine, Pirassununga, 13635-900, Brazil

[2]Technical University of Denmark, The National Veterinary Institute, Bygning 204, Lyngby, Denmark

[3]Agência Paulista de Tecnologia dos Agronegócios (APTA), Fisheries Institute, Sao Paulo, 05001-900, Brazil

***Corresponding author:** Dr. Marcelo Candido

Universidade de São Paulo (USP), Faculty of Animal Science and Food Engineering - Department of Veterinary Medicine

Email: marcelo.c@usp.br

Tel: +55 19 971309161

Fig. S1

Sequence	MH351268	KJ175144	AY548484	MF360246	JQ654586	KX185156	AF389451	KP266743	KX574341	MF538627	MF004271	KR075874	KR075886	KR075872	FJ433873	NC_028461	KT989885	KT989884	AY521625	NC_006549
MH351268 ←	100% ↑																			
KJ175144	99.26%	100%																		
AY548484	99.26%	100%	100%																	
MF360246	98.28%	96.79%	96.79%	100%																
JQ654586	98%	97.65%	97.65%	97.82%	100%															
KX185156	94.85%	94.98%	94.98%	95.62%	95.26%	100%														
AF389451	92.88%	93.05%	93.05%	94.1%	93.93%	93.53%	100%													
KP266743	39.24%	39.24%	39.24%	39.36%	39.25%	39.22%	38.27%	100%												
KX574341	39.53%	38.45%	38.45%	38.97%	39.28%	40%	38.19%	95.38%	100%											
MF538627	39.56%	38.67%	38.67%	38.95%	39.3%	39.97%	38.33%	95.84%	96.89%	100%										
MF004271	39.69%	38.59%	38.59%	39.11%	39.42%	40.16%	38.37%	96.12%	96.47%	97.77%	100%									
KR075874	50.49%	50.04%	50.04%	50.81%	50.33%	51.12%	51.94%	73.46%	74.67%	74.37%	74.44%	100%								
KR075886	50.52%	50.07%	50.07%	50.85%	50.35%	51.15%	51.98%	73.45%	74.58%	74.29%	74.38%	99.5%	100%							
KR075872	50.34%	50.15%	50.15%	50.81%	50.21%	50.93%	51.69%	73.33%	74.81%	74.51%	74.58%	99.5%	99.64%	100%						
FJ433873	54.27%	44.92%	44.92%	53.95%	54.05%	54.9%	55.45%	80.06%	79.53%	79.95%	79.54%	94.43%	94.36%	94.48%	100%					
NC_028461	54.27%	44.92%	44.92%	53.95%	54.05%	54.9%	55.45%	80.06%	79.53%	79.95%	79.54%	94.43%	94.36%	94.48%	100%	100%				
KT989885	55.57%	45.78%	45.78%	55.19%	55.26%	56.17%	56.52%	78.91%	78.41%	78.9%	78.48%	94.07%	93.99%	94.16%	89.68%	89.68%	100%			
KT989884	55.58%	45.72%	45.72%	55.19%	55.27%	56.18%	56.53%	78.92%	78.43%	78.91%	78.5%	94.09%	94.01%	94.18%	89.76%	89.76%	99.95%	100%		
AY521625	36.85%	27.64%	27.64%	36.49%	36.67%	37.23%	37.29%	38.35%	37.97%	38.03%	37.83%	39.71%	39.7%	39.75%	36.76%	36.76%	36.59%	36.54%	100%	
NC_006549	36.85%	27.64%	27.64%	36.49%	36.67%	37.23%	37.29%	38.35%	37.97%	38.03%	37.83%	39.71%	39.7%	39.75%	36.76%	36.76%	36.59%	36.54%	100%	100%

Supplementary Fig.1.
Identity of nucleotide sequences (%) of the complete genome of different species of *Ranavirus*.
The arrow in black indicates the sample obtained and analyzed in this study.

Fig. S2

Initial recombination breakpoint	Final recombination breakpoint	Potential major parent	Potential minor parent	Detection methods	*p* values
57535 bp	94377 bp	MF360246 - Frog virus 3 isolate Op/2015	None	RDP<br>GENECONV<br>Bootscan<br>MaxChi<br>Chimaera<br>SiScan<br>3Seq	9.879×10^{-51}<br>$3.99E \times 10^{-58}$<br>1.065×10^{-49}<br>8.983×10^{-04}<br>1.438×10^{-02}<br>1.217×10^{-43}<br>3.885×10^{-15}

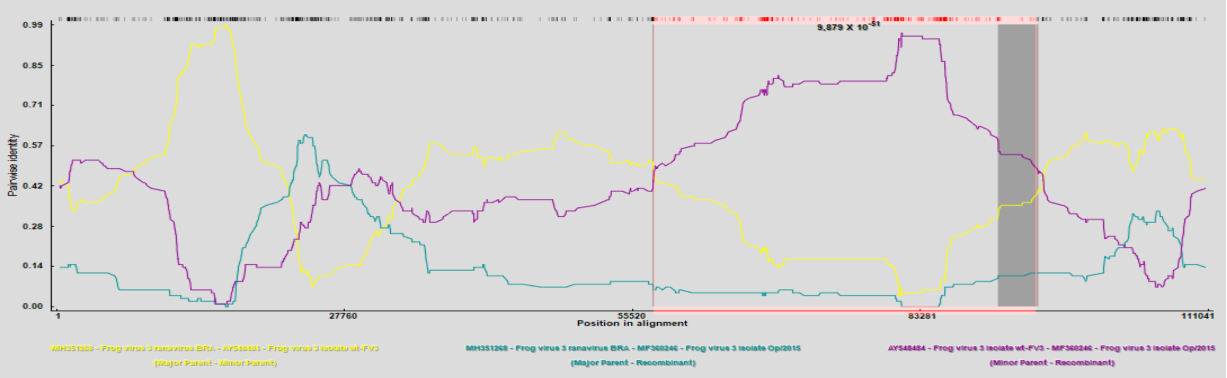

Initial recombination breakpoint	Final recombination breakpoint	Potential major parent	Potential minor parent	Detection methods	*p* values
78864 bp	81726 bp	AY548484 - Frog virus 3 isolate wt-FV3	AF389451 - Tiger frog virus	RDP<br>GENECONV<br>Bootscan<br>MaxChi<br>Chimaera<br>SiScan<br>3Seq	1.737×10^{-40}<br>2.502×10^{-24}<br>1.737×10^{-40}<br>1.408×10^{-17}<br>4.820×10^{-15}<br>1.043×10^{-14}<br>3.497×10^{-14}

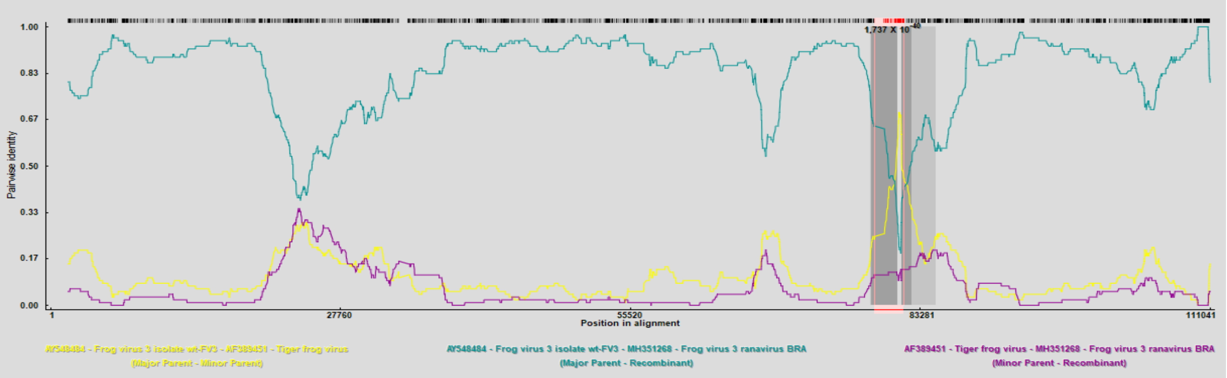

Initial recombination breakpoint	Final recombination breakpoint	Potential major parent	Potential minor parent	Detection methods	*p* values
22147 bp	30132 bp	AY548484 - Frog virus 3 isolate wt-FV3	None	RDP<br>GENECONV<br>Bootscan<br>MaxChi<br>Chimaera<br>SiScan<br>3Seq	6.172×10^{-10}<br>8.885×10^{-09}<br>1.142×10^{-29}<br>4.136×10^{-13}<br>2.899×10^{-14}<br>2.320×10^{-08}<br>3.885×10^{-15}

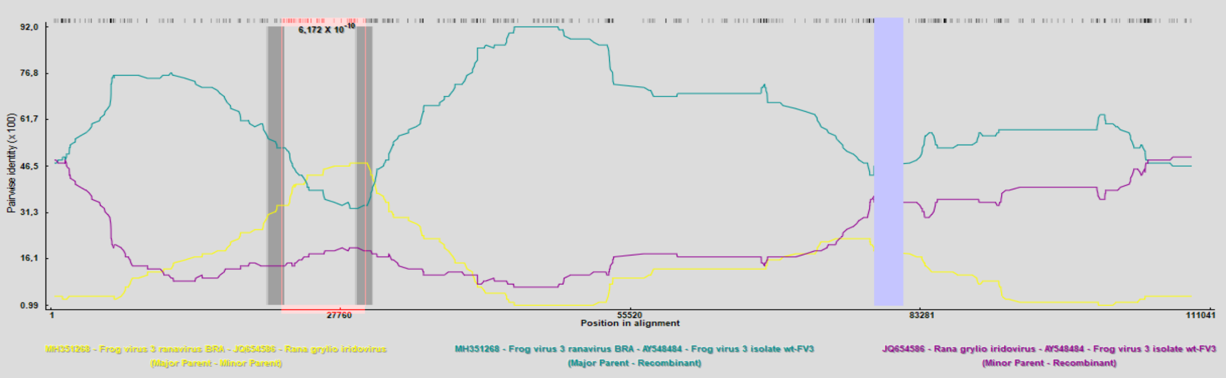

Initial recombination breakpoint	Final recombination breakpoint	Potential major parent	Potential minor parent	Detection methods	*p* values
85060 bp	86880 bp	AY548484 - Frog virus 3 isolate wt-FV3	None	RDP<br>GENECONV<br>Bootscan<br>MaxChi<br>Chimaera<br>SiScan<br>3Seq	9.757×10^{-11}<br>$3.57E \times 10^{-17}$<br>9.498×10^{-19}<br>8.346×10^{-09}<br>5.756×10^{-09}<br>1.655×10^{-16}<br>3.108×10^{-14}

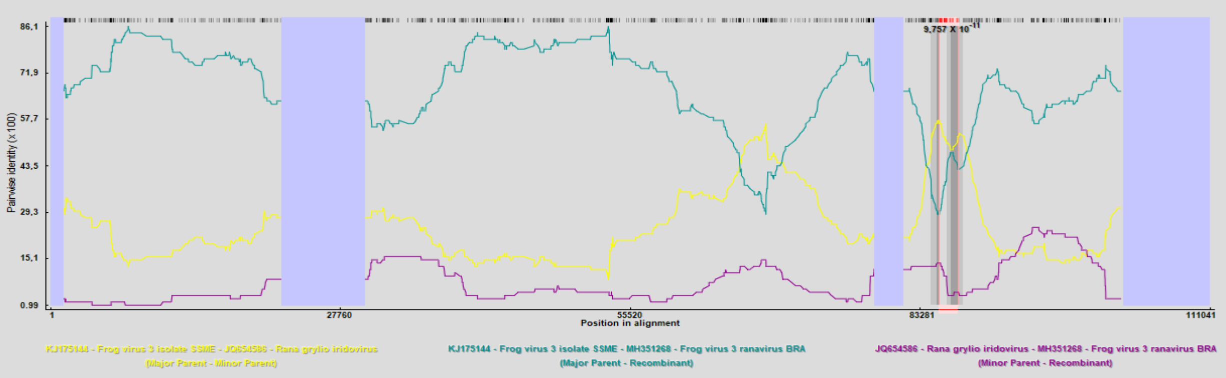

Initial recombination breakpoint	Final recombination breakpoint	Potential major parent	Potential minor parent	Detection methods	*p* values
65377 bp	69162 bp	KJ175144 - Frog virus 3 isolate SSME	JQ654586 - Rana grylio iridovirus	RDP<br>GENECONV<br>Bootscan<br>MaxChi<br>Chimaera<br>SiScan<br>3Seq	2.238×10^{-09}<br>1.347×10^{-06}<br>1.023×10^{-11}<br>5.370×10^{-09}<br>4.109×10^{-07}<br>1.232×10^{-16}<br>5.789×10^{-13}

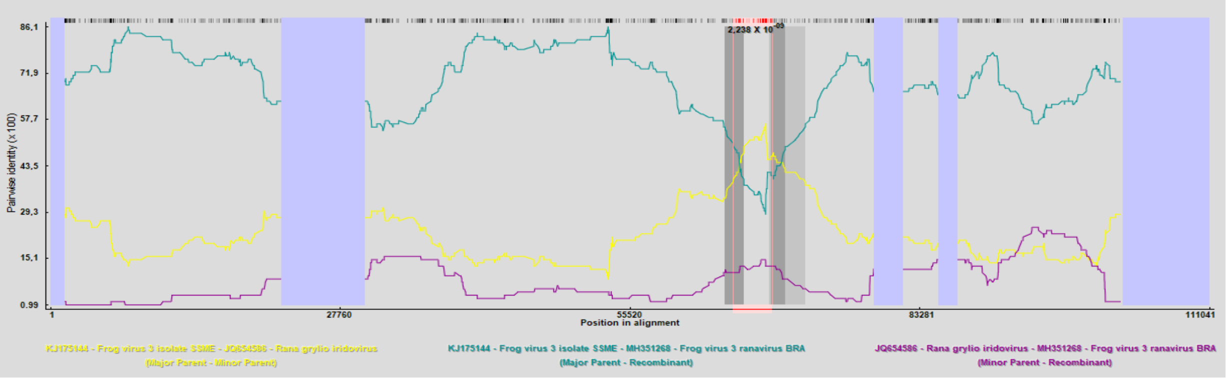

Initial recombination breakpoint	Final recombination breakpoint	Potential major parent	Potential minor parent	Detection methods	*p* values
15958 bp	18788 bp	MF360246 - Frog virus 3 isolate Op/2015	None	RDP<br>GENECONV<br>Bootscan<br>MaxChi<br>Chimaera<br>SiScan<br>3Seq	9.685×10^{-12}<br>3.570×10^{-04}<br>6.224×10^{-12}<br>8.505×10^{-10}<br>3.725×10^{-09}<br>2.438×10^{-15}<br>3.866×10^{-12}

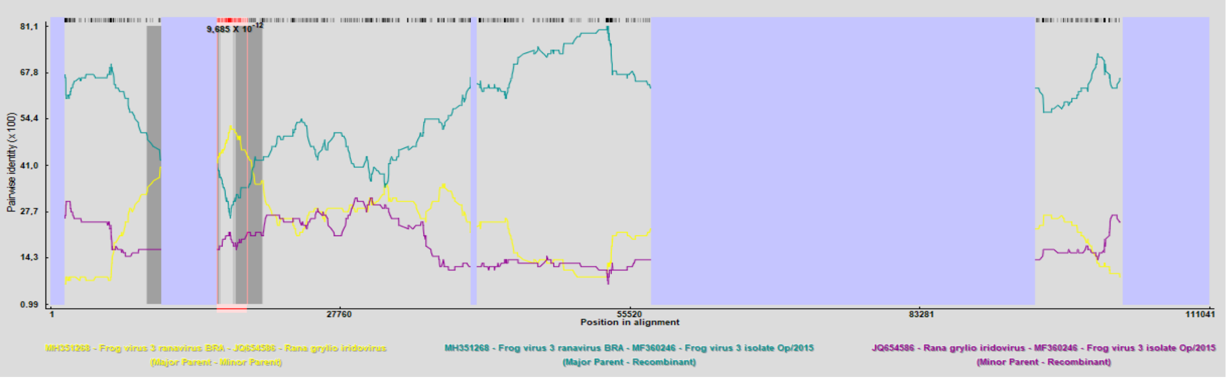

Initial recombination breakpoint	Final recombination breakpoint	Potential major parent	Potential minor parent	Detection methods	*p* values
1413 bp	1771 bp	JQ654586 - Rana grylio iridovirus	None	RDP<br>GENECONV<br>Bootscan<br>MaxChi<br>Chimaera<br>SiScan<br>3Seq	6.079×10^{-08}<br>2.236×10^{-08}<br>3.130×10^{-07}<br>----------------<br>3.663×10^{-02}<br>1.968×10^{-09}<br>7.042×10^{-05}

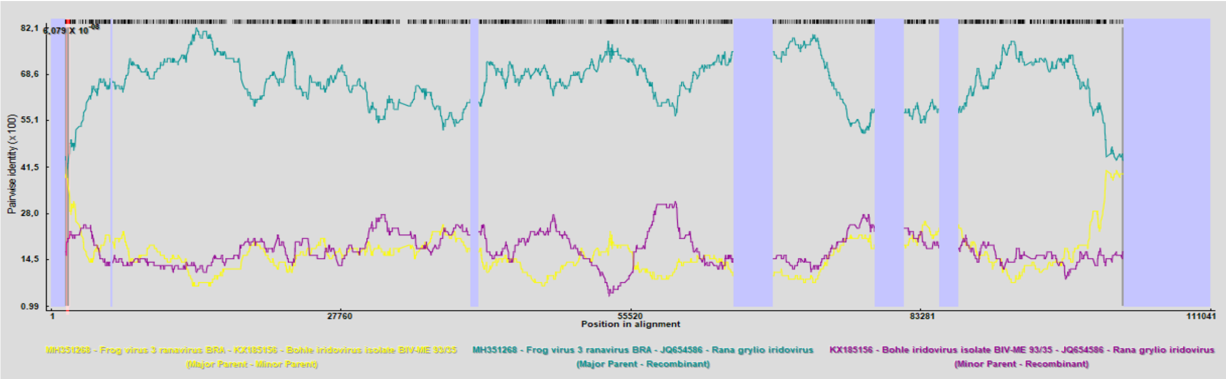

Initial recombination breakpoint	Final recombination breakpoint	Potential major parent	Potential minor parent	Detection methods	*p* values
100911 bp	101480 bp	None	KX185156 - Bohle iridovirus isolate BIV-ME 93/35	RDP<br>GENECONV<br>Bootscan<br>MaxChi<br>Chimaera<br>SiScan<br>3Seq	----------------<br>1.092×10^{-06}<br>1.907×10^{-07}<br>1.485×10^{-02}<br>----------------<br>7.433×10^{-05}<br>1.909×10^{-03}

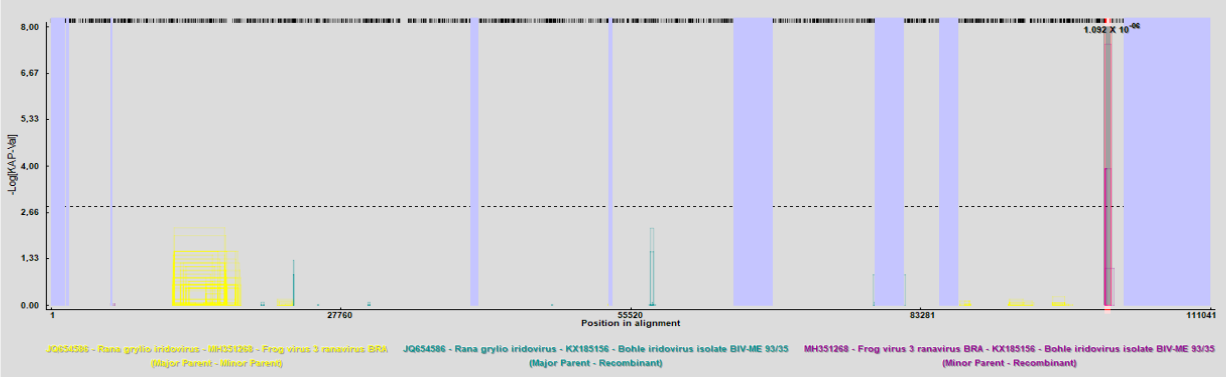

Supplement: Supplementary file 1 — Supplementary Figures [file 41598_2019_53626_MOESM1_ESM.pdf]
